# Single-cell RNA sequencing reveals cellular dynamics and therapeutic effects of astragaloside IV in slow transit constipation

**DOI:** 10.17305/bb.2024.10187

**Published:** 2024-08-01

**Authors:** Huaxian Chen, Xingyang Wan, Qiulan He, Guozhong Xiao, Yihui Zheng, Minyi Luo, Chaoxin Yang, Donglin Ren, Li Lu, Hui Peng, Hongcheng Lin

**Affiliations:** 1 Department of General Surgery (Department of Coloproctology), The Sixth Affiliated Hospital, Sun Yat-sen University, Guangzhou, China; 2 Guangdong Provincial Key Laboratory of Colorectal and Pelvic Floor Diseases, The Sixth Affiliated Hospital, Sun Yat-sen University, Guangzhou, China; 3 Biomedical Innovation Center, The Sixth Affiliated Hospital, Sun Yat-sen University, Guangzhou, China; 4 Department of Anaesthesiology, First Affiliated Hospital of Sun Yat-sen University, Guangzhou, China

**Keywords:** Slow transit constipation (STC), astragaloside IV (AS-IV), single-cell RNA sequencing (scRNA-seq), cellular dynamic, intestinal cells

## Abstract

The cellular characteristics of intestinal cells involved in the therapeutic effects of astragaloside IV (AS-IV) for treating slow transit constipation (STC) remain unclear. This study aimed to determine the dynamics of colon tissue cells in the STC model and investigate the effects of AS-IV treatment by single-cell RNA sequencing (scRNA-seq). STC mouse models were developed using loperamide, with subsequent treatment using AS-IV. Colon tissues and feces were collected for scRNA-seq and targeted short-chain fatty acid quantification. We integrated scRNA-seq data with network pharmacology to analyze the effect of AS-IV on constipation. AS-IV showed improvement in defecation for STC mice induced by loperamide. Notably, in STC mice, epithelial cells, T cells, B cells, and fibroblasts demonstrated alterations in cell proportions and aberrant functions, which AS-IV partially rectified. AS-IV has the potential to modulate the metabolic pathway of epithelial cells through its interaction with peroxisome proliferator-activated receptor gamma (PPARγ). AS-IV reinstated fecal butyrate levels and improved energy metabolism in epithelial cells. The proportion of naïve CD4+T cells is elevated in STC, and the differentiation of these cells into Treg is regulated by B cells and fibroblasts through the interaction of ligand–receptor pairs. AS-IV treatment can partially alleviate this trend. The status of fibroblasts in STC undergoes alterations, and the FB_C4_Adamdec1 subset, associated with angiogenesis and the Wingless-related integration (Wnt) pathway, emerges. Our comprehensive analysis identifies perturbations of epithelial cells and tissue microenvironment cells in STC and elucidates mechanisms underlying the therapeutic efficacy of AS-IV.

## Introduction

Chronic constipation is a prevalent gastrointestinal tract disorder with a reported incidence ranging from 6.9% to 14.9% [1, 2]. Categorized by pathogenesis, chronic constipation is primarily divided into three types: slow transit constipation (STC), outlet obstruction constipation, and mixed constipation. Among these, STC accounts for a substantial proportion of cases [3]. Presently, the complex pathogenesis of STC is not yet fully understood, leading to a variety of treatments with inconsistent efficacies. As a result, developing a standardized and effective diagnostic and therapeutic approach has proven challenging. Therefore, further clarification of the pathogenesis of STC and the identification of more efficacious diagnostic and therapeutic approaches are critical to advancing the field.

STC is linked to the enteric nervous system, which includes changes in enteric nerve cells, damage to intestinal glial cells, and alterations in neurotransmitters. Additionally, immune and endocrine factors, psychological influences, and lifestyle changes, such as diet and exercise habits, are also considered contributory. Nonetheless, there is limited research on how epithelial and stromal cells within the intestinal tissues are affected in STC.

Astragaloside IV (AS-IV) has been listed as the main active component of *Astragalus mongholicus* Bunge [4]. According to previous research, AS-IV exerts various functions, including anti-inflammatory, anti-fibrotic, anti-oxidative, anti-asthmatic, anti-diabetic, and immunomodulatory activities [5]. In our prior research, AS-IV was effective in the treatment of the STC model induced by loperamide [6]. To further understand the complex and heterogeneous mechanisms of STC, we created an STC mouse model using loperamide, conducted single-cell RNA sequencing (scRNA-seq) to explore changes in the STC lesion microenvironment, and investigated the mechanism of AS-IV treatment for STC.

## Materials and methods

### Animal model

We acquired KM mice, aged 6–8 weeks, from Guangzhou Chashi Ruihua Biotechnology Co., LTD., China, to perform the animal experiments. The mice were randomly divided into four groups: Control group (CON, *n* ═ 8), STC (*n* ═ 8), AS-L (*n* ═ 8), and AS-H (*n* ═ 8). Ten days post-modeling, intestinal transit tests were conducted on different groups (three mice from each group), and colon tissues were collected for single-cell isolation (two mice from CON, STC, and AS-H groups). Additionally, fresh fecal samples from the colon (three samples from CON, STC, and AS-H groups) were gathered and stored at –80 ^∘^C for subsequent analysis.

### Modeling and administration

Except for the CON group, all groups received 10 mg/kg of loperamide (Aladdin, CAS: 34552-83-5) twice daily via oral gavage for five consecutive days to establish an STC model. The CON group was administered an equivalent volume of sterile water. Following successful modeling, the STC group continued to receive loperamide and 10 g/L sodium carboxymethyl cellulose via oral gavage for the next five consecutive days. To assess AS-IV’s efficacy in treating STC, the AS-L and AS-H groups were administered loperamide and either a low dose (10 mg/kg) or a high dose (90 mg/kg) of AS-IV, respectively, over the subsequent five days [6]. AS-IV was sourced from Shanghai Aladdin Biochemical Technology Co., Ltd., China, with a purity exceeding 98% (Batch number: A274907; CAS: 83207-58-3).

### Measurement of intestinal charcoal transit ratio

On the 10th day of the experiment, mice were subjected to a 12-h fast without water deprivation. Subsequently, activated charcoal (0.2 g/mL) was added to the drug solution via oral gavage. Thirty minutes later, the mice were euthanized humanely, and their abdominal cavities were meticulously opened. The intestinal tract was dissected from the pylorus to the ileocecal valve and laid out on a whiteboard. The total length of the intestinal tract (L1) and the transit distance from the pylorus to the leading edge of the ink mark (L2) were measured. The intestinal transit ratio was calculated using the formula L2/L1*100% [7].

### Western blotting

Total proteins from mouse colon tissues were extracted using RIPA Lysis Buffer (Servicebio, Cat: G2002) per the manufacturer’s instructions. Protein concentrations were determined using a BCA assay kit (Servicebio, Cat: G2026). Proteins were separated by 10% SDS-PAGE, transferred to PVDF membranes (Millipore, Cat: IPVH00010), and blocked with 5% lipid-free milk for 1 h. Membranes were incubated with primary antibodies overnight at 4 ^∘^C, washed with TBST, and then incubated with secondary antibodies for 1 h at room temperature. A chemiluminescent substrate (Servicebio, Cat: G2161) was used for detection. β-actin (Servicebio, Cat: GB15003) served as an internal standard, with the primary antibody targeting PPARγ (Santa Cruz, Cat: sc-7273).

### Sample collection and single-cell suspension preparation

The fresh colon tissue was washed with cold PBS, chopped on ice, and enzymatically digested. The enzyme mixture contained 0.1 mL of 20-mg/mL collagenase D (Roche, Cat: 11088858001) and 0.3 mL of 10-mg/mL Dispase II (Roche, Cat: 04942078001) per 0.2 g of tissue. Incubation occurred for 60 min at 37 ^∘^C with shaking at 150 rpm. Post-dissociation, the cell suspension was filtered through a 70-µm cell strainer, and centrifuged at 300×*g* for 7 min at 4 ^∘^C. The supernatant was removed, and red blood cells lysed as needed. The cell pellet was resuspended in cold 0.04% BSA/PBS for scRNA-seq. Cells were stained with AO/PI and assessed for viability using a Countstar Fluorescence Cell Analyzer.

### Single-cell library preparation and sequencing

The transcriptomic information of single cells was captured using the BD Rhapsody system. The workflow of BD Rhapsody single-cell whole-transcriptome amplification was followed to prepare the whole transcriptome libraries. A high-sensitivity DNA chip (Agilent) on a Bioanalyzer 2200 and the Qubit High Sensitivity DNA assay (Thermo Fisher Scientific) was used to quantify the libraries. Sequencing was performed by the Illumina sequencer (Illumina, San Diego, CA, USA) on a 150-bp paired-end run.

### Quantification, quality control, and cluster analysis of single-cell expression

After obtaining the raw sequencing data, we used fastp with default parameters to filter the adaptor sequence and remove low-quality reads. Unique molecular identifier (UMI) tools were then utilized for single-cell transcriptome analysis to identify the cell barcode whitelist. The UMI-based clean data was mapped to the mouse genome reference using STAR mapping with customized parameters from the UMI-tools standard pipeline to obtain the UMI counts of each sample. Seurat package (version: 4.3.0, https://satijalab.org/seurat/) in R software was used for quality control and further analysis [8]. Cells with fewer than 200 or more than 6000 expressed genes or a mitochondrial UMI rate exceeding 30% were excluded. Data were normalized and scaled, focusing on the 2000 high-variable genes. PCA was constructed based on the scaled data. The top 20 principal components were then used for tSNE construction. Utilizing the graph-based cluster method, we used tSNE to visualize data in two dimensions. The differentially expressed genes (DEGs) for clusters were calculated by the FindAllMarkers function with default parameters. Major cell type was annotated with canonical marker genes. To further identify the specific cell type, the clusters of each cell type were selected for re-tSNE analysis, graph-based clustering, and marker analysis.

### Differentially expressed genes

The FindMarkers function was used to identify the DEGs between two groups. The DEGs were filtered based on the following criteria: Wilcoxon Rank Sum test, min. *P*ct ═ 0.1, *P* value < 0.05, |logfc.threshold| > 0.25.

### Gene ontology (GO) enrichment analysis

To perform GO enrichment analysis, the top 200 genes exhibiting differential expression were filtered by fold change and analyzed using the clusterProfiler R package [9]. The results from biological processes (BPs) of the enrichment analysis were selected based on a statistical threshold (qvalueCutoff ═ 0.05).

### scMetabolism analysis

scMetabolism (v0.2.1) is an R package that calculates the score of the certain metabolic pathway of each cell [10]. In our study, the epithelial cells were used to compare metabolic disparities between the three groups. We used the ssGSEA quantification method in this package to evaluate the metabolic score of each epithelial cell in the reactome pathway [11]. The result was a *z*-score transformed to produce a heatmap.

### The comparison of pathway

The geneset pathway was acquired from MSigDB [11]. We computed the average expression level of the geneset in each cell. Subsequently, all cells were divided into three groups (CON, STC, and AS group) and a comparison was conducted between the groups, which was visualized using a violin plot.

### Trajectory analysis using Monocle 2

Monocle 2 was used to elucidate the transcriptional dynamics among cell types [12]. Briefly, we create a CellDataSet object in the default setting. The variable genes were obtained by differentialGeneTest function and filtered with qval < 0.01. The DDRTree method and the orderCells function were utilized for dimensional reduction and cell ordering.

### Cell–cell communication analysis

CellChat (https://github.com/sqjin/CellChat) was utilized to analyze the cell–cell interactions among different cell types [13]. This R package can quantitatively calculate the intercellular communication networks and predict the primary signal pathways, which were then used to visualize the signaling pathway networks. Only ligands and receptors expressed in at least 10% of specific cells were considered for use for analysis.

### Screening of AS-IV targets for constipation treatment

SMILES identifier of AS-IV was retrieved from the PubChem database [CC1(C(CCC23C1C(CC4C2(C3)CCC5(C4(CC(C5C6(CCC(O6)C(C)(C)O)C)O)C)C)OC7C(C(C(C(O7)CO)O)O)O)OC8C(C(C(CO8)O)O)O)C]–(https://pubchem.ncbi.nlm.nih.gov/) – where the downloaded SDF file is. Potential targets for AS-IV were obtained utilizing the PharmMapper, and targets with *z*-scores > 0 were selected and retained for further analysis [14]. Additionally, the targets of the active ingredients were predicted by submitting the SMILES identifier for AS-IV to Swiss Target Prediction, with potential genes being identified as targets having a Prob value > 0 [15]. Subsequently, all potential target names were converted to their respective official gene names in the UniProt database. The target genes obtained from both databases were integrated and processed, with duplicate genes being removed. “Constipation” was used as the keyword to obtain constipation-related genes from GeneCards and DisGeNET databases. The screening conditions were set at GeneCards’ Score > 0.05 and DisGeNET’s Score > 0.1. Ultimately, the disease-associated genes were processed and integrated, with any duplicate genes being eliminated.

### Construction of protein–protein interaction (PPI) network

To determine the interaction targets of AS-IV that may be pertinent to the treatment and pathology of constipation, we utilized the ggVennDiagram package to generate a Venn diagram. The overlapping targets were subsequently uploaded onto the STRING (Version 12.0) platform (https://string-db.org/) to acquire a PPI network (high confidence, 0.70; species, Mus musculus) [16]. The string interactions file was exported from the STRING website and then imported into Cytoscapse software (v3.10.1) for visualization. The CytoHubba plug-in’s BottleNeck algorithm was utilized to perform a comprehensive analysis of network topology, which enabled us to identify the top 10 hub genes.

### Targeted short-chain fatty acid (SCFA) quantitation

The feces samples were homogenized with 500 µL of water and 100 mg of glass beads for 1 min. After centrifugation at 4 ^∘^C for 10 min at 12,000 rpm, 200 µL of the supernatant was extracted using a mixture of 100 µL of 15% phosphoric acid, 20 µL of 375 µg/mL 4-methylvaleric acid solution as the internal standard, and 280 µL of ether. Following vortexing for 1 min, the samples were centrifuged again at 4 ^∘^C for 10 min at 12,000 rpm, and the resulting supernatant was transferred into a vial for subsequent GC-MS analysis. Gas chromatography-mass spectrometry analysis of SCFA in the colonic contents of mice was performed by BioNovoGene Technology Ltd. (Suzhou, China). The GC analysis was performed on a trace 1300 gas chromatograph (Thermo Fisher Scientific, USA). Mass spectrometric detection of metabolites was performed on ISQ 7000 (Thermo Fisher Scientific, USA).

### Analysis of fecal microbiota composition through 16S DNA sequencing

The feces sample’s genomic DNA was extracted using either the CTAB or SDS method. The DNA’s purity and concentration were assessed through agarose gel electrophoresis. Polymerase chain reaction (PCR) was then performed using diluted genomic DNA as a template, with primers targeting the 16S V4 region (515F and 806R). The PCR products underwent electrophoresis on a 2% agarose gel, and target bands were purified using Qiagen’s gel recovery kit. Library construction was carried out using the TruSeq^®^ DNA PCR-Free Sample Preparation Kit. Libraries were quantified using Qubit and Q-PCR, and sequencing was executed on the NovaSeq6000 platform after library qualification.

### Ethical statement

The Animal Care and Use Committee of Ruiye Bio-tech Guangzhou Co., Ltd., approved all animal experiments under License No. RYEth-20221123109.

### Statistical analysis

Statistical data were analyzed using R software (v4.1.2) and GraphPad (v8.3.0). The data quantifications were reported as mean ± standard deviation. Statistical analyses of the data were performed using either a one-way ANOVA with Dunnett’s multiple comparisons test for comparisons among three or more groups, or a two-tailed Student’s *t*-test for comparisons between two groups. To assess significance, a value of *P* < 0.05 was considered statistically significant (*P < 0.05; **P < 0.01; ***P < 0.001; ****P < 0.0001).

## Results

### AS-IV ameliorates defecation in STC mice induced by loperamide

**Figure 1.**
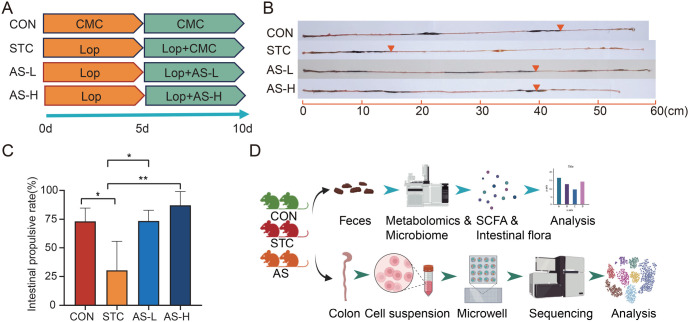
**AS-IV ameliorates defecation in STC mice induced by loperamide.** (A) A schematic representation of the establishment of the STC model and treatment with AS-IV; (B) The effects of AS-IV treatment on intestinal charcoal transit in loperamide-induced STC mice; (C) The quantitative comparison of the intestinal transit charcoal ratio between the CON, STC, AS-L, and AS-H groups. A *P* value < 0.05 was considered statistically significant (each group: *n* ═ 3); (D) Schematic overview of experimental design and analytical workflow. *P < 0.05; **P < 0.01. CON: Control group; STC: Group of slow transit constipation model; AS-L: Group of treatment of low dose of AS-IV; AS-H: Group of treatment of high dose of AS-IV; Lop: Loperamide; CMC: Carboxymethyl cellulose sodium; AS-IV: Astragaloside IV. Figures were generated with BioRender (https://biorender.com/).

The study involved four groups of mice: CON, STC, AS-L, and AS-H. We established an STC mouse model induced by loperamide, and the AS-L and AS-H groups received treatment with low and high doses of AS-IV (Figure 1A). Compared to the CON group, the intestinal charcoal transit ratio was significantly reduced in the STC group (Figure 1B and 1C). The changes in transit ratio confirmed the successful establishment of the STC mice model. The administration of AS-IV to loperamide-induced STC mice resulted in improved defecation, suggesting that AS-IV can ameliorate STC induced by loperamide (Figure 1B and 1C).

To investigate the dynamics of colon epithelial cells and tissue microenvironment cells, as well as fecal microflora and metabolites in the STC model and AS-IV-treated mice, we conducted both scRNA-seq of colon tissue, 16S DNA Sequencing and targeted SCFA quantitation of fecal samples. A total of six fresh colon tissue samples were obtained from mice, with two samples being collected from each of the following groups: CON, STC, and AS group. Following the preparation of single-cell suspensions, we utilized the BD Rhapsody system to perform single-cell capture and cDNA library preparation. Next, a digital expression matrix was generated from the UMI counts for each gene in each cell. In addition, fecal samples were collected from the mice and subjected to targeted SCFA quantitation and 16S DNA Sequencing. These data were used as input for further bioinformatic analysis (Figure 1D).

### scRNA-seq23 demonstrates the reshaping of tissue microenvironment induced by AS-IV in STC mice

**Figure 2.**
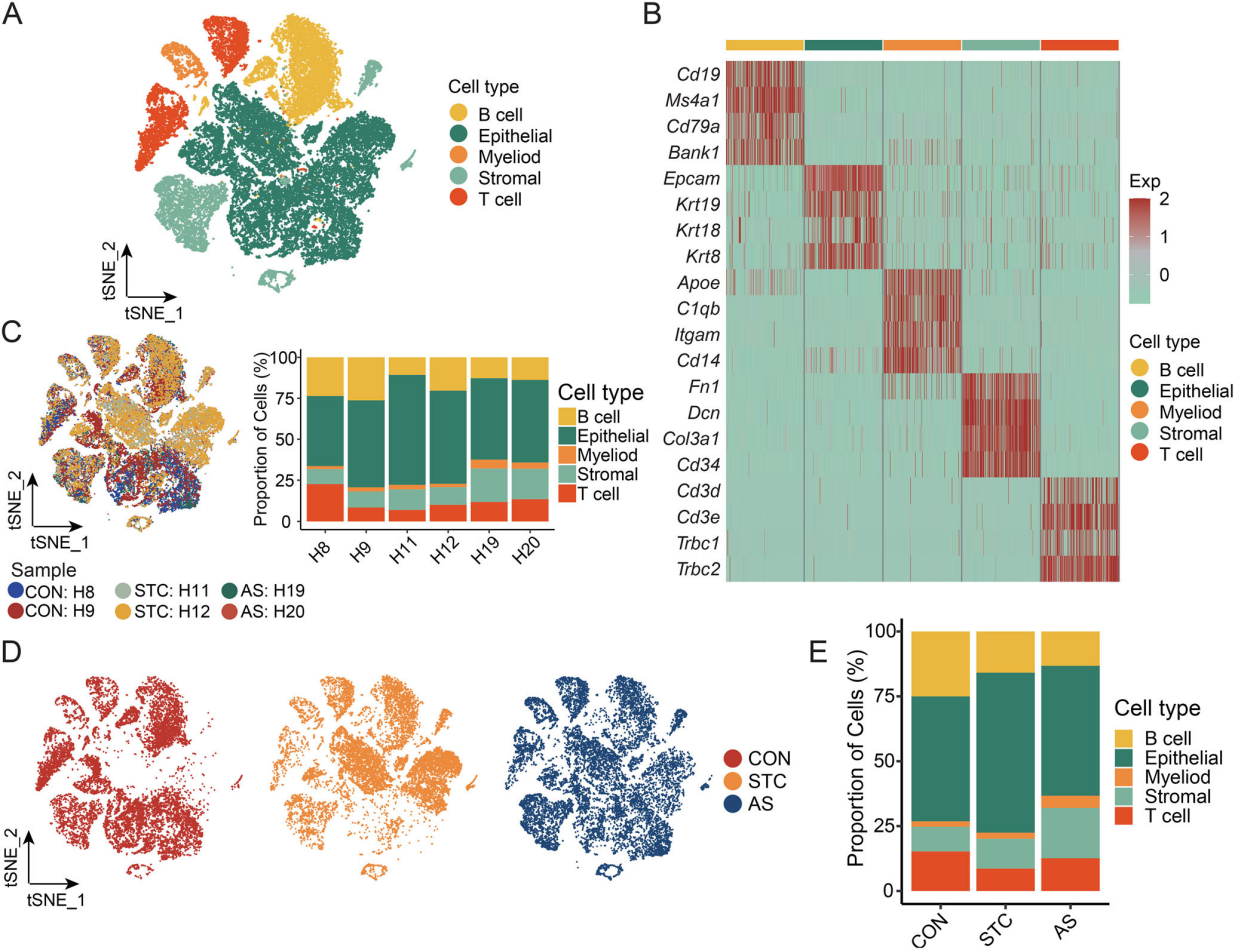
**scRNA-seq demonstrates the reshaping of tissue microenvironment induced by AS-IV in STC mice.** (A) t-SNE plot of the major cell types. There are 39,305 cells after quality control from six samples; (B) The expression of the canonical marker genes of the major cell types; (C) t-SNE plots and stacked bar charts display the distribution of major cell types among the six samples; (D) The t-SNE plot illustrates the dynamic changes of major cell types between the CON, STC, and AS groups; (E) The stacked bar chart illustrates the dynamic changes of major cell types between the CON, STC, and AS groups. CON: Control group; STC: Group of slow transit constipation model; AS: Group of treatment with AS-IV; AS-IV: Astragaloside IV; scRNA-seq: Single-cell RNA sequencing.

**Figure S1.**
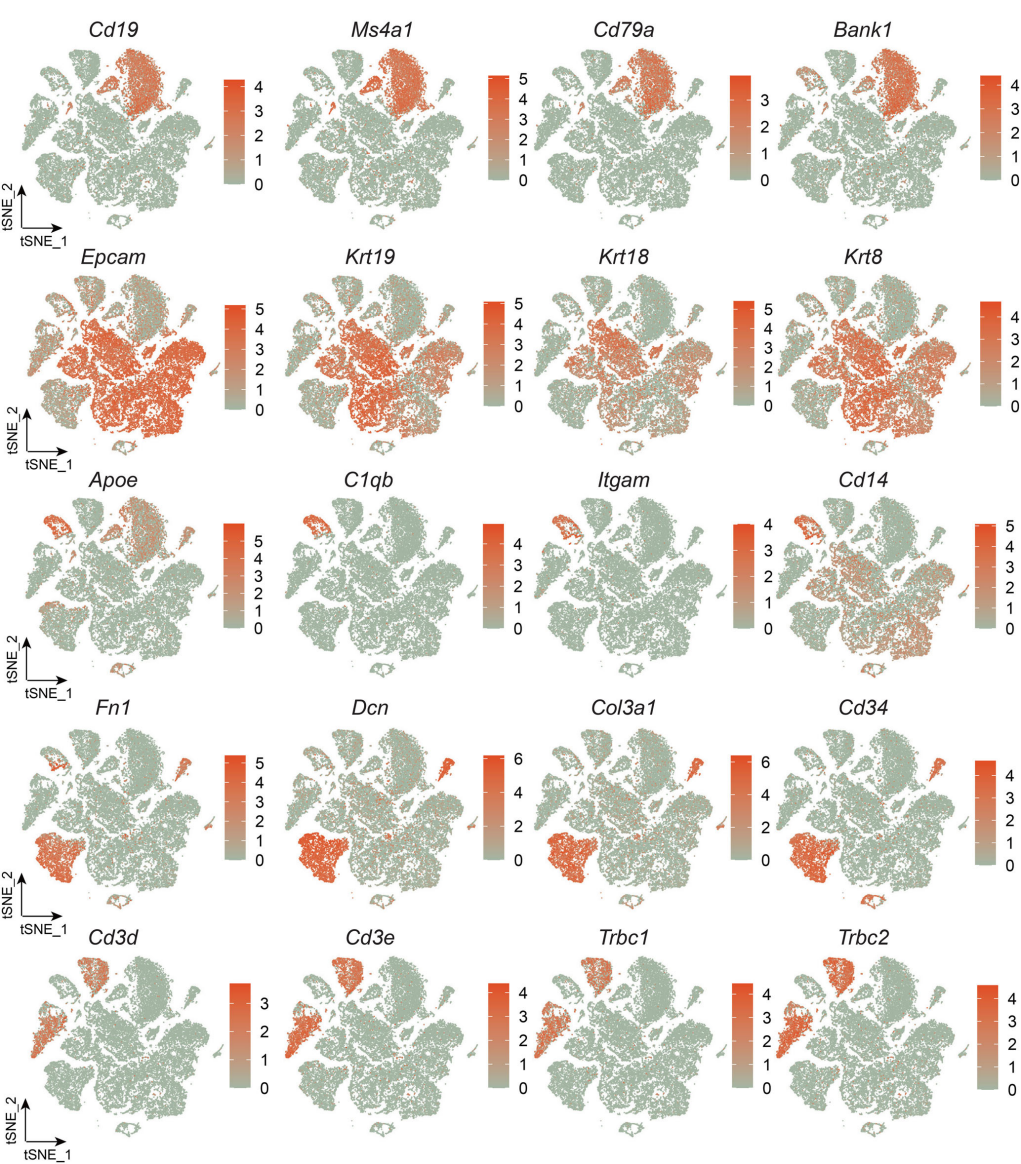
**scRNA-seq demonstrates the reshaping of tissue microenvironment induced by AS-IV in STC mice.** The t-SNE plot of expression of marker genes of each cell type. STC: Group of slow transit constipation model; AS-IV: Astragaloside IV; scRNA-seq: Single-cell RNA sequencing.

Following Seurat-based quality control and filtration to our scRNA-Seq data, we obtained a final dataset comprising 39,305 cells. These included 13,605 cells from the CON group, 12,694 from the STC group, and 13,006 from the AS group. Canonical marker genes helped identify five major cell types: epithelial cells (*Epcam, Krt19*), stromal cells (*Fn1, Dcn*), T cells (*Cd3d, Trbc1*), B cells (*Cd19, Ms4a1*), and myeloid cells (*C1qb, Itgam*) (Figures 2A and 2B and S1A). The prevalence of each cell type remained consistent across all samples (Figure 2C). Notable transcriptional heterogeneity was observed among these cell types across the three groups (Figure 2D), with variations in cell type proportions when comparing the groups (Figure 2E). This data indicated significant remodeling in B cells, epithelial cells, stromal cells, and T cells within the AS group. Consequently, scRNA-seq has revealed changes in the tissue microenvironment of STC mice induced by AS-IV.

### AS-IV regulates the functional status of epithelial cells

**Figure 3.**
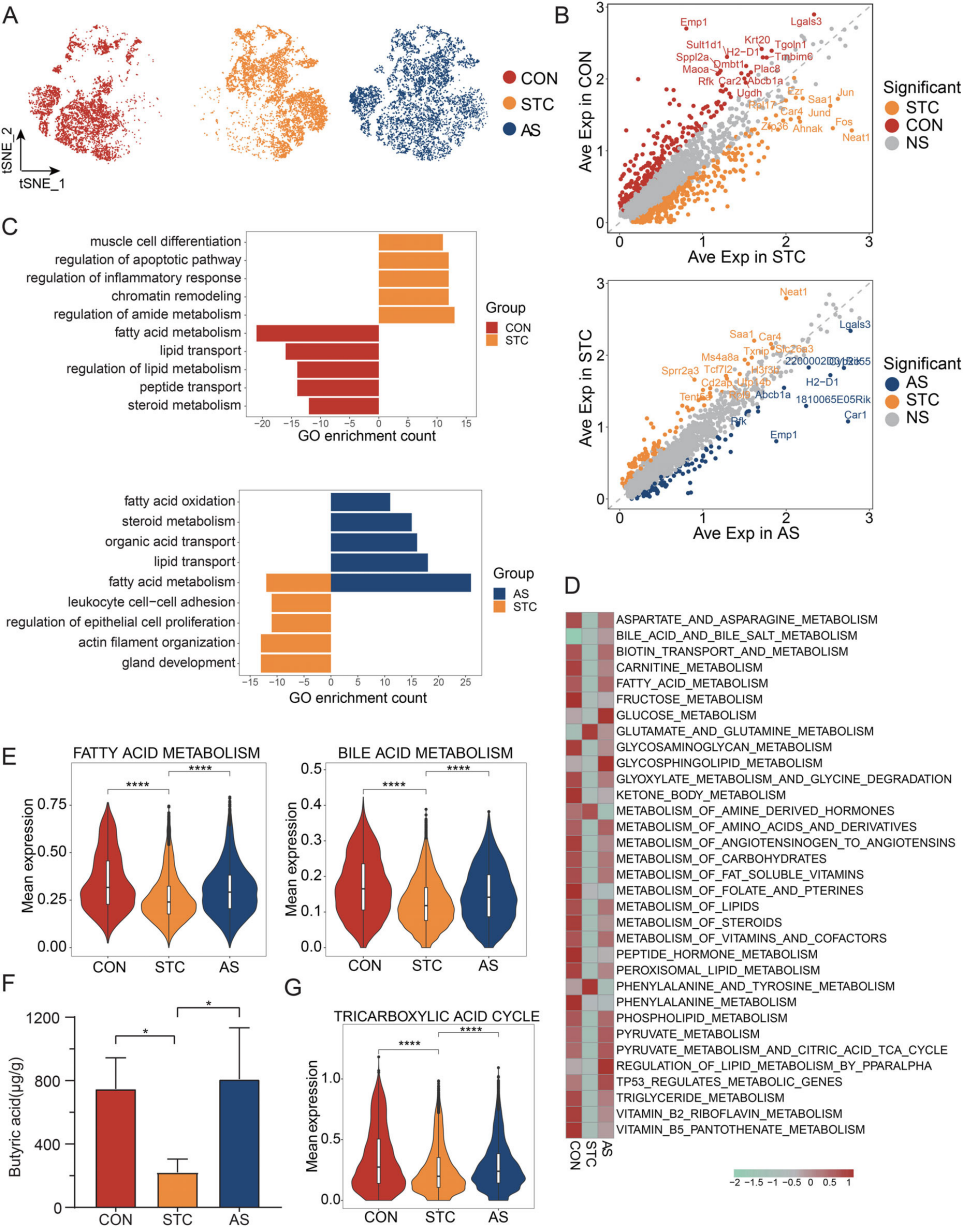
**AS-IV regulates the functional status of epithelial cells.** (A) The t-SNE plot illustrates the dynamic changes of epithelial cells among the CON, STC, and AS groups; (B) DEGs of epithelial cells. Top: DEGs between STC and CON. Orange dot: DEGs in CON group; Red dot: DEGs in STC group. Bottom: DEGs between AS and STC. Orange dot: DEGs in STC group; Blue dot: DEGs in AS group. DEGs were defined as those with a *P* value < 0.05 from the Wilcoxon rank sum test and a log2 fold change > 0.25; (C) Barplot of GO enrichment (BPs) of the epithelial cells based on DEGs. Top: GO enrichment based on DEGs between STC and CON; Bottom: GO enrichment based on DEGs between AS and STC. The length of the bars represents the count of genes enriched in the pathway. Only gene sets with *q* values < 0.05 were plotted; (D) The heatmap displays the metabolic pathway score of epithelial cells among the CON, STC, and AS groups. the metabolic pathway from the Reactome pathway; (E) Violin plot of the fatty acid metabolism (left) and bile acid metabolism (right) pathway expression in the epithelial cells; (F) The comparison of the butyric acid between CON, STC, and AS groups. A *P* value < 0.05 (* mark) was considered as statistically significant; (G) Violin plot of the tricarboxylic acid cycle pathways expression in the epithelial cells. *P < 0.05; **P < 0.01; ***P < 0.001; ****P < 0.0001. CON: Control group; STC: Group of slow transit constipation model; AS: Group of treatment with AS-IV; AS-IV: Astragaloside IV; DEG: Differentially expressed genes; GO: Gene ontology; NS: Not significant; Ave Exp: Average expression.

Chronic constipation is known to impair the intestinal mucosal barrier, with colonic epithelial cells playing a pivotal role in maintaining this barrier [17]. We detected significant changes in the transcriptomic profiles of epithelial cells in both the STC and AS groups (Figure 3A) and observed noticeable variations in their proportions (Figure 2E). To delve deeper into these functional shifts, we identified upregulated DEGs in both the STC and AS groups (Figure 3B). Subsequently, GO enrichment analysis was then conducted to explore changes in BPs within epithelial cells across the three groups (Figure 3C). The analysis revealed that, compared to the CON and AS group, epithelial cells in the STC group were significantly involved in inflammatory responses, regulation of epithelial cell proliferation, and leukocyte cell–cell adhesion. In contrast, the AS group showed enrichment in fatty acid metabolism, lipid transport, and steroid metabolism pathways compared to the STC group. To further elucidate these findings, we compared metabolic gene set changes among the three groups using the scMetabolism package (Figure 3D). Our results indicated a general decrease in metabolic pathway scores in the STC group, with a restoration of these scores observed in the AS group.

**Figure S2.**
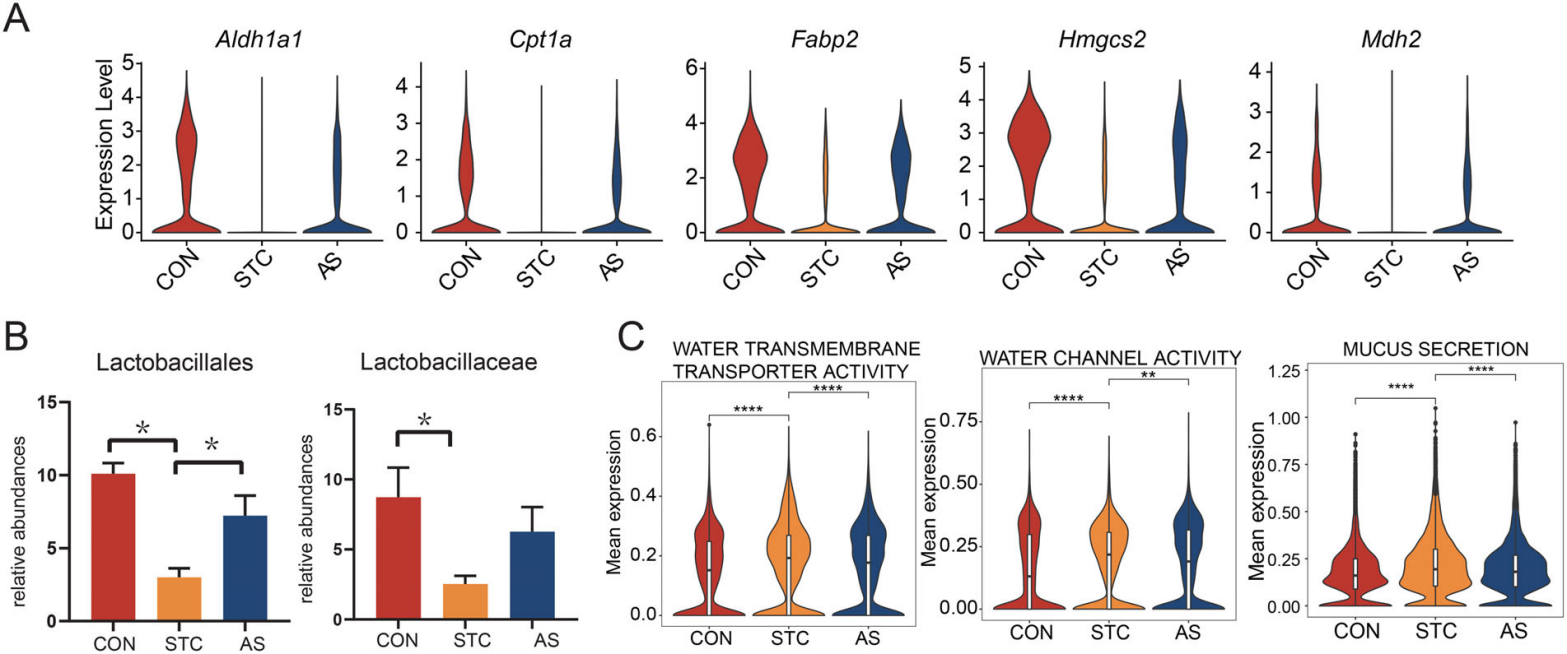
**AS-IV regulates the functional status of epithelial cells.** (A) Violin plot of the relevant genes associated with fatty acid metabolism in the epithelial cells; (B) The comparison of relative abundances of order *Lactobacillales* (left) and Family *Lactobacillaceae* (right) in mouse feces between CON, STC, and AS groups. A *P* value < 0.05 (* mark) was considered as statistically significant; (C) Violin plot of the water transmembrane transporter activity (left), water channel activity (middle), and mucus secretion (right) pathway expression in the epithelial cells. CON: Control group; STC: Group of slow transit constipation model; AS: Group of treatment with AS-IV; AS-IV: Astragaloside IV.

Considering the close relationship between STC and the metabolic processes of fatty acids and bile acids facilitated by gut microorganisms, we emphasize the role of epithelial cells as key components of the intestinal mucosal barrier, interacting with and influenced by these metabolic products. We evaluated the gene sets related to epithelial cells across the three groups and found that bile acid and fatty acid metabolism in epithelial cells were reinstated in the AS group (Figure 3E). Key genes linked to fatty acid metabolism, such as *Aldh1a1*, *Cpt1a*, *Fabp2*, *Hmgcs2*, and *Mdh2*, exhibited a consistent pattern (Figure S2A).

**Figure 4.**
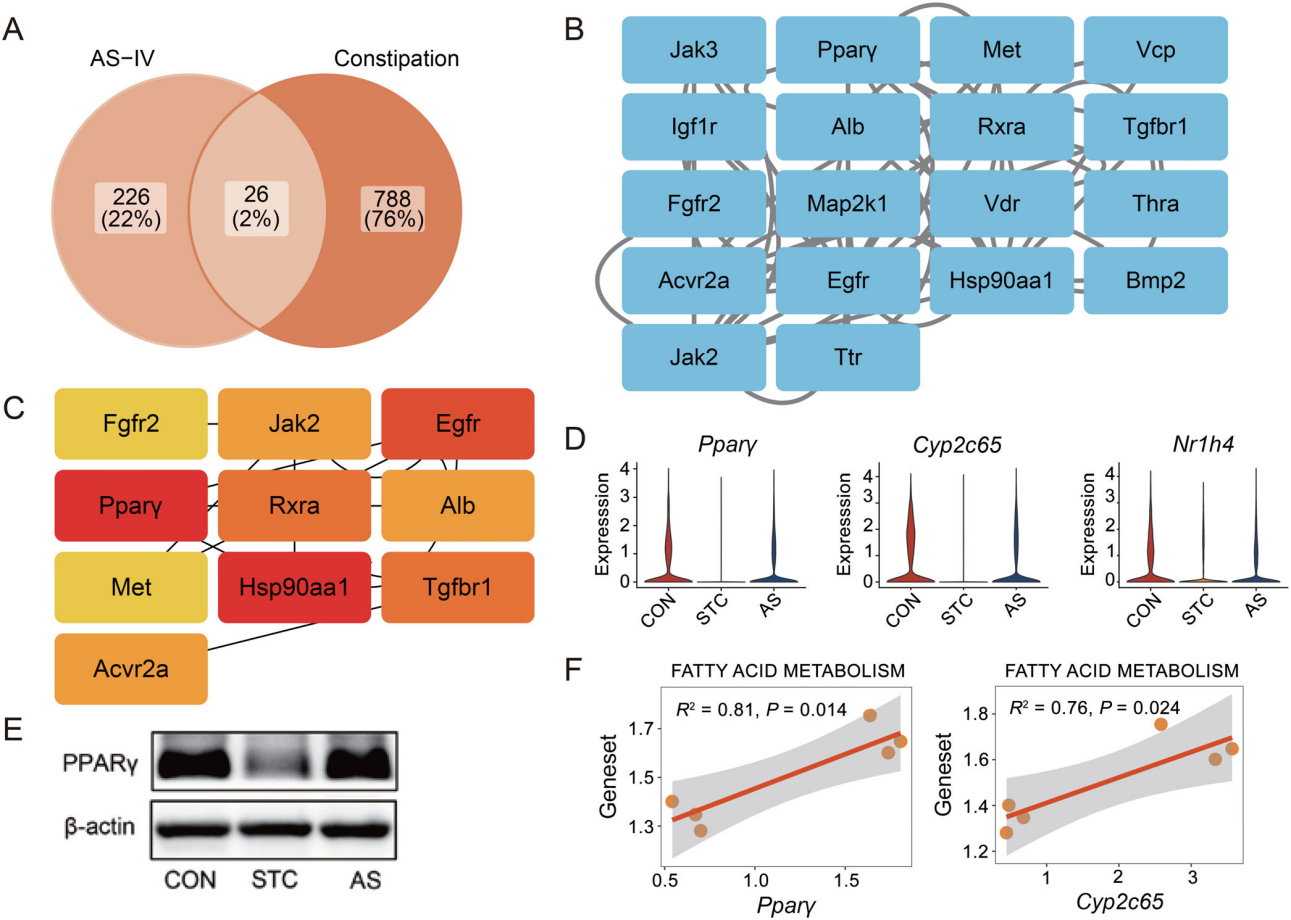
**PPI network of candidate AS-IV targets for STC treatment.** (A) Venn diagram showing the overlap among AS-IV targets (left) and constipation-related genes (right). A total of 26 genes were identified as common; (B) PPI network of potential targets of AS-IV in the treatment of constipation; (C) Top 10 core target genes identified by the Cytobubba; (D) Violin plot of AS-IV target gene expression in the epithelial cells. Left: *Pparγ*; Middle: *Cyp2c65*; Right: *Nr1h4*; (E) Relative levels of PPARγ proteins in mouse colon tissue. β-actin was tested as the internal standard; (F) Scatterplot shows the correlation between two genes (*Pparγ* and *Cyp2c65*) and fatty acid metabolism pathway gene sets in the epithelial cells. PPI: Protein–protein interactions; PPARγ: Proliferator-activated receptor gamma; CON: Control group; STC: Group of slow transit constipation model; AS: Group of treatment with AS-IV; AS-IV: Astragaloside IV.

Analysis of targeted SCFA quantitation in fecal samples indicated a reduction in butyrate levels in the STC group, which was restored in the AS group (Figure 3F). Additionally, 16S DNA Sequencing analysis revealed a decline in the order *Lactobacillales* and family *Lactobacillaceae* in the STC group, which was recovered in the AS group (Figure S2B). Butyrate, a byproduct of gut microbiota metabolism, is a crucial energy source for epithelial cells, as reported in literature [18]. At low concentrations, butyrate undergoes β-oxidation in mitochondria, yielding acetyl-CoA, which initiates the tricarboxylic acid cycle to energize intestinal epithelial cells. The comparison of tricarboxylic acid cycle pathways across the three groups supports this mechanism (Figure 3G). Furthermore, considering STC’s association with intestinal water and mucus secretion, we note that epithelial cells play a role in both absorption and secretion. Our findings indicate that water transport and mucus secretion pathways in the STC group were significantly altered, but the AS group could partially rectify these changes (Figure S2C). These results suggest that STC prompts metabolic shifts in epithelial cells, while AS-IV treatment helps restore these disrupted pathways.

### PPI network of AS-IV targets for STC treatment

AS-IV is a bioactive, small-molecule organic compound that serves as the primary active ingredient in traditional Chinese medicine *Astragalus mongholicus* Bunge (Huangqi) [19]. To elucidate the specific mechanism by which AS-IV regulates the molecular pathways in epithelial cells, we conducted a comprehensive analysis of the Swiss target prediction database and the PharmMapper database, identifying 30 and 224 potential target genes for AS-IV, respectively. The two target gene lists were merged, and duplicates were removed, to generate 252 AS-IV target genes. Furthermore, we conducted a screening of constipation-related genes in the GeneCards database and the DisGeNET database, resulting in 809 and 23 genes, respectively. After removing duplicate values, a total of 814 genes were identified. The intersection of the AS-IV-related target genes and the constipation-related genes yielded 26 common genes (Figure 4A), representing potential gene targets regulated by AS-IV for constipation.

We utilized the STRING database to obtain the PPI network of the potential gene targets regulated by AS-IV for constipation. And the network which represented the interactions between the target genes was visualized by Cytoscapse (Figure 4B). To further screen for key genes in the network, the Cytoscape plugin CytoHubba was utilized, resulting in the identification of the top ten key targets, including Fgfr2, Jak2, Egfr, Pparγ, Rxra, Alb, Met, Hsp90aa1, Tgfbr1, and Acvr2a (Figure 4C). Given the association of constipation with aquaporins and SCFA [20, 21], we focused on PPARγ, which is involved in fatty acid release, transport, and storage such as lipoprotein lipase and the fatty acid transporter CD36 [22], and a regulator of lipid metabolism and an identified key gene in the AS-IV target network (Figure 4B and 4C). P*parγ* expression was notably decreased in the STC group compared to others (Figure 4D), aligning with reduced fatty acid metabolism pathway scores (Figure 3E). Western blot analysis confirmed PPARγ inhibition in the STC group (Figure 4E). *Pparγ* expression correlated significantly with fatty acid metabolism pathways (Figure 4F), suggesting that AS-IV may enhance fatty acid metabolism via *Pparγ* activation. Other AS-IV targets like *Cyp2c65* and *Nr1h4* also showed differential expression (Figure 4D). CYP2C65 is generally found in mouse colon tissue and is involved in fatty acid metabolism pathways [23, 24]. The expression of *Cyp2c65* was downregulated in the STC group, while it was restored in the AS group. And the correlation of *Cyp2c65* expression and fatty acid metabolism pathway also suggested that AS-IV may have multiple targets to regulate fatty acid metabolism (Figure 4F). NR1H4 (Farnesoid X receptor) is involved in bile acid metabolism [25]. Altered bile acid metabolism has been reported to play a role in the pathophysiology of constipation [26, 27]. Overall, our findings suggest that AS-IV may act on multiple target proteins to regulate metabolic pathways effectively.

### Alterations in T cell subset status

**Figure 5.**
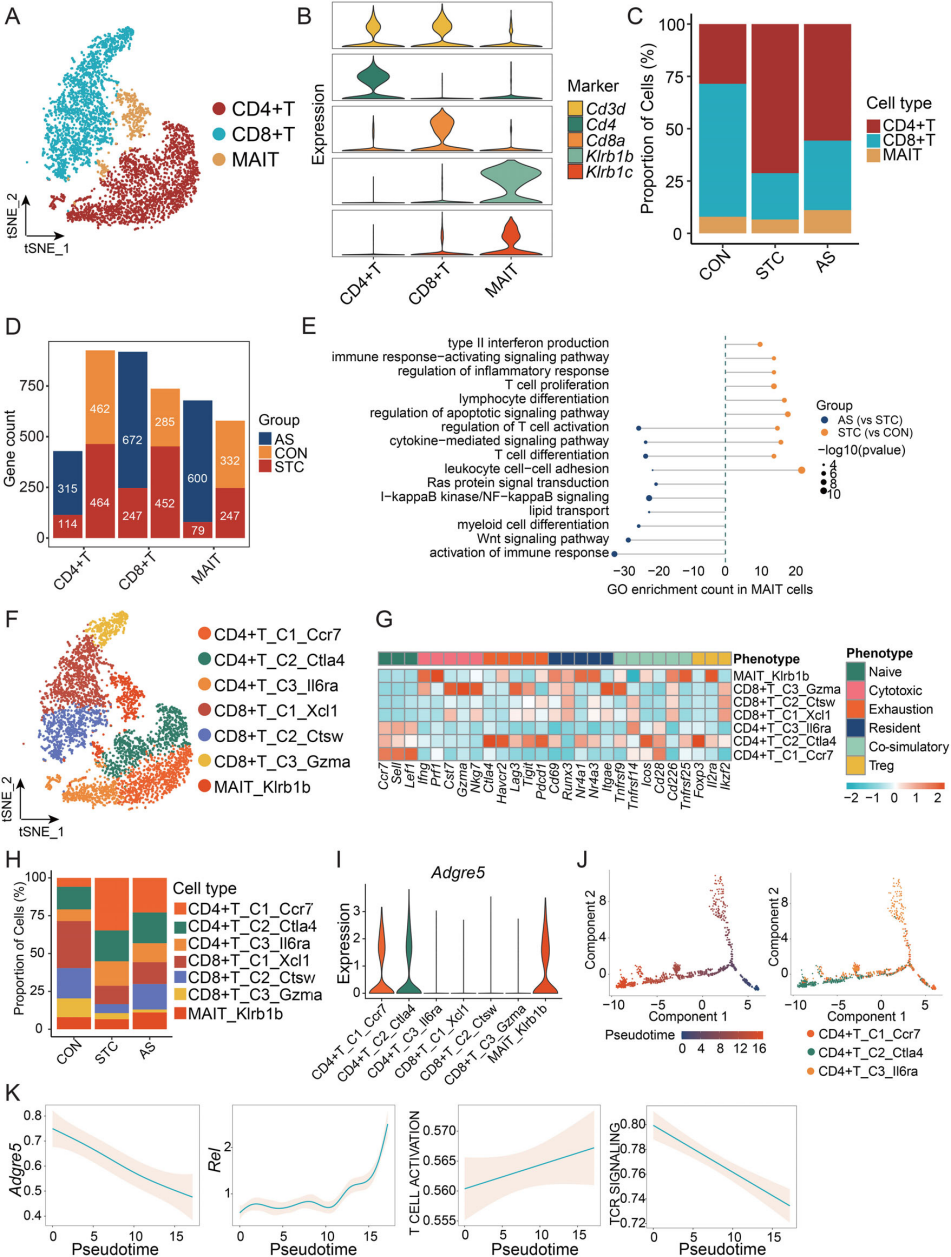
**Alterations in T cell subset status.** (A) The t-SNE plot of major T cell subsets; (B) Violin plot of marker gene expression in major T cell subsets; (C) The stacked bar chart illustrates the dynamic changes of major T cell subsets between the CON, STC, and AS groups; (D) The number of DEGs of major T cell subsets. Orange: DEGs in CON group; Red: DEGs in STC group; Blue: DEGs in AS group. DEGs were defined as those with a *P* value < 0.05 from the Wilcoxon Rank Sum test and a log2 fold change > 0.25; (E) GO enrichment (BPs) of the MAIT cells based on DEGs. Left: GO enrichment of AS compared with STC; Right: GO enrichment of STC compared with CON. The length of the bars represents the count of genes enriched in the pathway. Only gene sets with *q* values < 0.05 were plotted; (F) The t-SNE plot of T cell subtypes; (G) Heatmap displays the expression of selected gene sets in T cell subtypes, including naive, cytotoxic, exhaustion, resident, co-stimulatory, and Treg marker genes; (H) The stacked bar chart illustrates the dynamic changes of T cell subtypes among the CON, STC, and AS groups; (I) Violin plot of *Adgre5* genes expression in the T cell subtypes; (J) Pseudotime trajectory analysis of CD4+ T cells. Left: Trajectory is colored by pseudotime; Right: Trajectory is colored by CD4+ T cell subtypes; (K) The expression dynamics of *Agdre5, Rel,* T cell activation, and TCR signaling pathway geneset across the pseudotime. CON: Control group; STC: Group of slow transit constipation model; AS: Group of treatment with AS-IV; AS-IV: Astragaloside IV; DEG: Differentially expressed genes; GO: Gene ontology; MAIT: Mucosal-associated invariant T.

**Figure S3.**
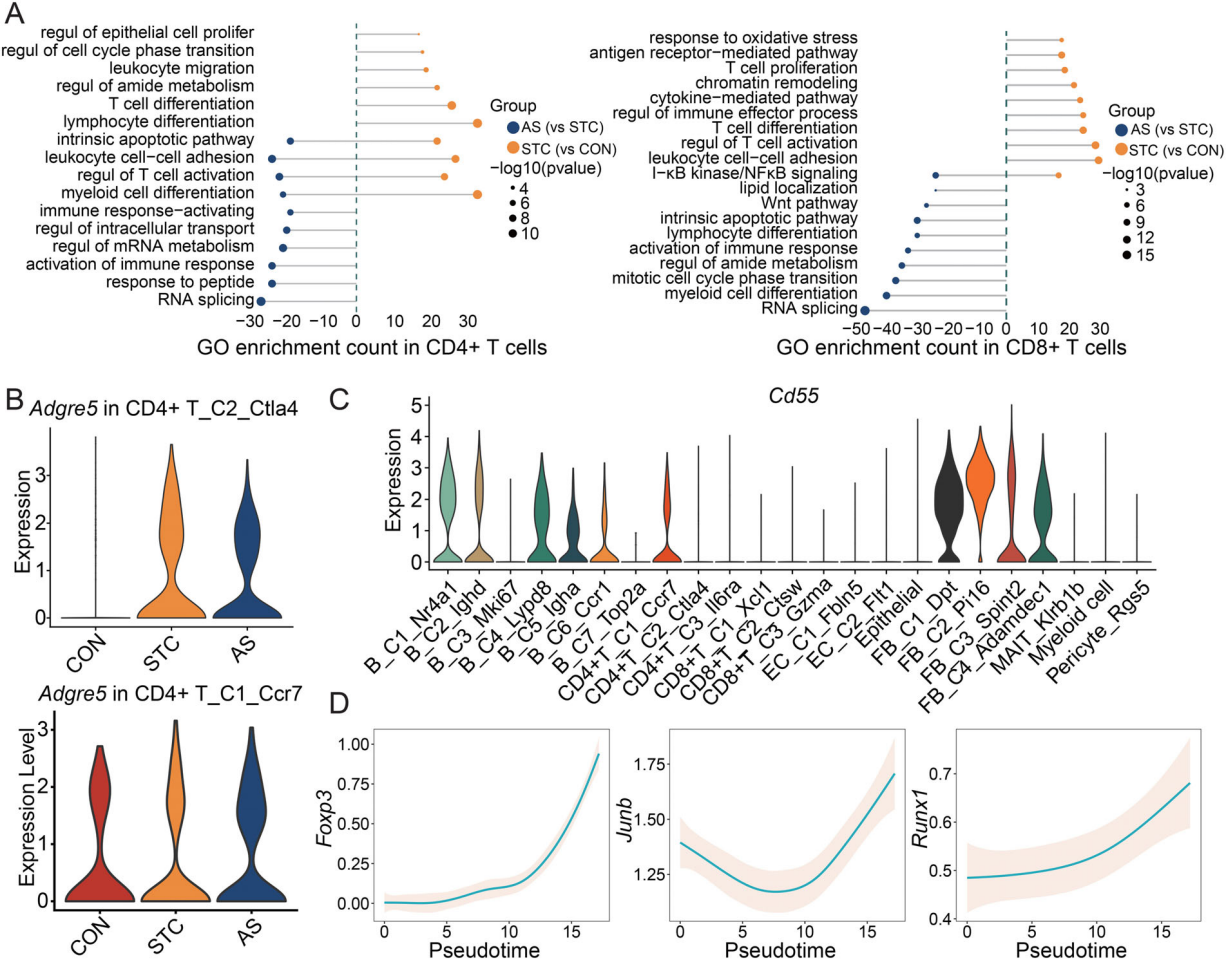
**Alterations in T cell subset status.** (A) GO enrichment (BPs) of the CD4+ T cells and CD8+ T cells based on DEGs. Left: GO enrichment of AS compared with STC; Right: GO enrichment of STC compared with CON. The length of the bars represents the count of genes enriched in the pathway. Only gene sets with *q* values < 0.05 were plotted; (B) Violin plot of the *Adgre5* expression in the CD4+ T_C2_Ctla4 and CD4+ T_C1_Ccr7; (C) Violin plot of the *Cd55* expression in the whole cell subtypes; (D) The expression dynamics of *Foxp3, Junb, and Runx1* across the pseudotime. Regul: Regulation; Prolifer: Proliferation; CON: Control group; STC: Group of slow transit constipation model; AS: Group of treatment with AS-IV; AS-IV: Astragaloside IV; GO: Gene ontology.

Research on the intestinal immune system of STC is limited. We examined the dynamics in T cell subsets, as they are important components of specific immunity and play a critical role in the inflammatory process. We first identified three major T cell subsets including CD4+ T cells, CD8+ T cells, and mucosal-associated invariant T (MAIT) cells (Figure 5A and 5B). The proportion of T cell subtypes (CD4+ T, CD8+ T, and MAIT) varied across groups (Figure 5C). DEG analysis was conducted to determine the differences in gene expression patterns between the AS and STC groups, as well as between the STC and CON groups (Figure 5D). More than 300 genes were upregulated in the major T cell subtypes of the AS group. The pathways upregulated in MAIT cells of the STC model were mainly related to cytokine-mediated signaling pathway and regulation of inflammatory response. The pathways upregulated in MAIT cells after AS-IV treatment were mainly related to the Wnt signal pathway and Ras protein signal transduction (Figure 5E). The results from GO analysis show that the upregulated genes in CD4+ T cells of the STC model were related to lymphocyte differentiation, leukocyte migration, and regulation of epithelial cell proliferation. The upregulated genes in CD8+ T cells of the STC model were related to T cell proliferation, regulation of T cell activation, and cytokine-mediated pathway (Figure S3A).

**Figure 6.**
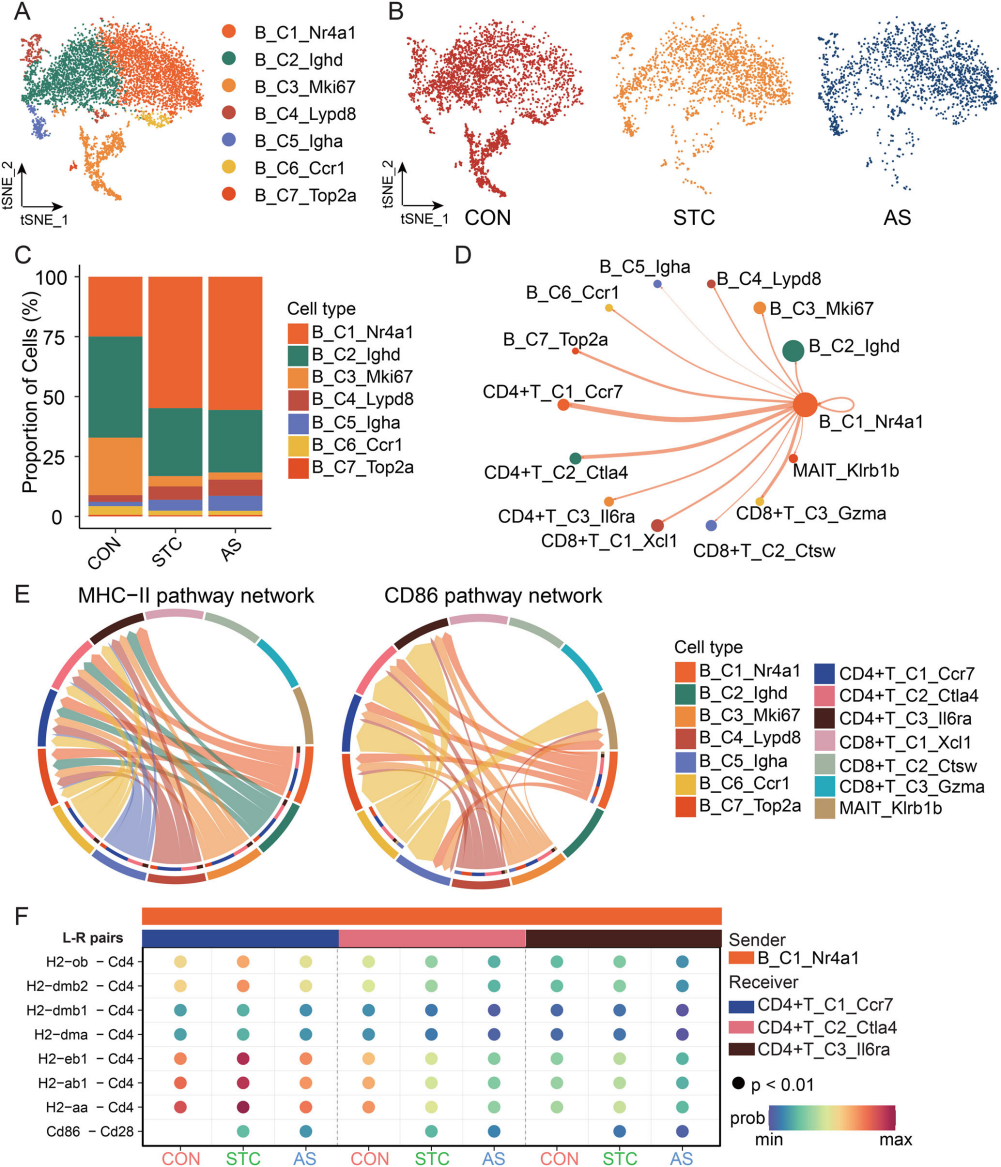
**B cells involvement in the regulation of T cells.** (A) The t-SNE plot of B cell subtypes; (B) The t-SNE plot illustrates the dynamic changes of B cells among the CON, STC, and AS groups; (C) The stacked bar chart illustrates the dynamic changes of B cell subtypes between the CON, STC, and AS groups; (D) B_C1_Nr4a1 ligand–receptor interaction network diagram. The size of the outer circle color indicates the number of cells; the cells emitting the arrow indicate the ligand and the cells pointing to the receptor; the edge width indicates the interaction strength (weights); (E) Chord diagram showing the network of MHC-II signaling pathways (left) and CD86 signaling pathways mediated only by Cd86-Cd28 (right) in T cells and B cells subtypes; (F) The comparison of communication probabilities from B_C1_Nr4a1 to CD4+ T cell subtypes among the CON, STC, and AS groups. CON: Control group; STC: Group of slow transit constipation model; AS: Group of treatment with AS-IV; AS-IV: Astragaloside IV.

CD4+ T cells were classified into three clusters, namely CD4+ T_C1_Ccr7, CD4+ T_C2_Ctla4, and CD4+ T_C3_Il6ra (Figure 5F and 5G). The proportions of these clusters displayed differences across the three groups (Figure 5H). CD4+ T_C1_Ccr7 significantly expresses marker genes of naïve T cells (Figure 5G), suggesting an increase in naïve CD4+ T cells in STC, and the treatment of AS-IV can reduce naïve CD4+ T cell infiltration. Previous research demonstrated that the binding of ligand CD55 to receptor ADGRE5 (CD97) can prompt the differentiation of naïve T cells into Treg-like cells [28, 29]. CD4+ T_C1_Ccr7 and CD4+ T_C2_Ctla4 express *Adgre5* (*Cd97*), and the *Adgre5* expression is higher in CD4+ T_C2_Ctla4 from the STC group (Figures 5I and S3B). We also investigated the origin of the ligand *Cd55* and observed that it is primarily derived from B cells and fibroblasts (Figure S3C). This implies that naïve CD4+T cells in STC might have been prompted to differentiate into Treg cells by more potent stimulation, ultimately resulting in augmented immunosuppression. Therefore, we employed trajectory analysis to investigate the differentiation of CD4+ T cells (Figure 5J). The expression of *Adgre5* is higher during the initial phase and gradually diminishes along the pseudotime (Figure 5K). The transcription factors (*Rel, Foxp3, Junb, and Runx1*) that are associated with Treg development and differentiation gradually increase along the pseudotime (Figures 5K and S3D). Furthermore, we found that T cell activation pathways gradually increase, while TCR pathways gradually decrease along pseudotime, which was consistent with the trend of *Adgre5* (Figure 5K).

Similarly, three CD8+ T cell subtypes were classified (Figure 5F and 5G). The proportion of CD8+ T_C1_Xcl1 and CD8+ T_C2_Ctsw in the AS group was higher than that in the STC group (Figure 5H).

The observations suggest that T cells in STC exhibit an immunosuppressed state, whereas AS-IV treatment leads to partial recovery. Consequently, in part, the therapeutic efficacy of AS-IV on STC can be attributed to its capacity to modulate T cell proportions and functionalities.

### B cells involved in the regulation of T cells

B cells serve as the primary effector cells of humoral immunity and can act as antigen-presenting cells to modulate T-cell function. B cells in the STC group demonstrate notable alterations. In the STC group, there is an increase in the population of B_C1_Nr4a1, alongside a decrease in the population of B_C2_Ighd (Figure 6A–6C). This finding implies that B_C1_Nr4a1 may play roles in disease initiation. The B_C1_Nr4a1 exhibits more robust interactions with CD4+ T cells (Figure 6D). MHC Class II molecules can be canonically recognized by CD4+ T cells. B_C1_Nr4al communicates with CD4+ T cells via the MHC-II and CD86 pathways (Figure 6E). Different communication probabilities of ligand-receptor interaction were observed between B cells and CD4+ T cells among the three groups. The communication probabilities of the interaction of H2 genes (*H2-eb1, H2-ab1, and H2-aa*) – *Cd4* were higher in the STC group than in the CON and AS group (Figure 6F). The ligand CD86 binds to the receptor CD28 to provide costimulatory signals to CD4+ T cells. Specifically targeting CD28 activation via CD86 has been shown to promote the proliferation and survival of Treg cells [30]. STC group exhibited a communication probability of the interaction between *Cd86* and *Cd28*, whereas the CON group was not present (Figure 6F). These findings suggest that B cells within the STC group exhibit alterations and are involved in the modulation of T-cell function through ligand–receptor interactions.

### Dynamic variation in fibroblast subtypes in STC and AS-IV treatment

**Figure 7.**
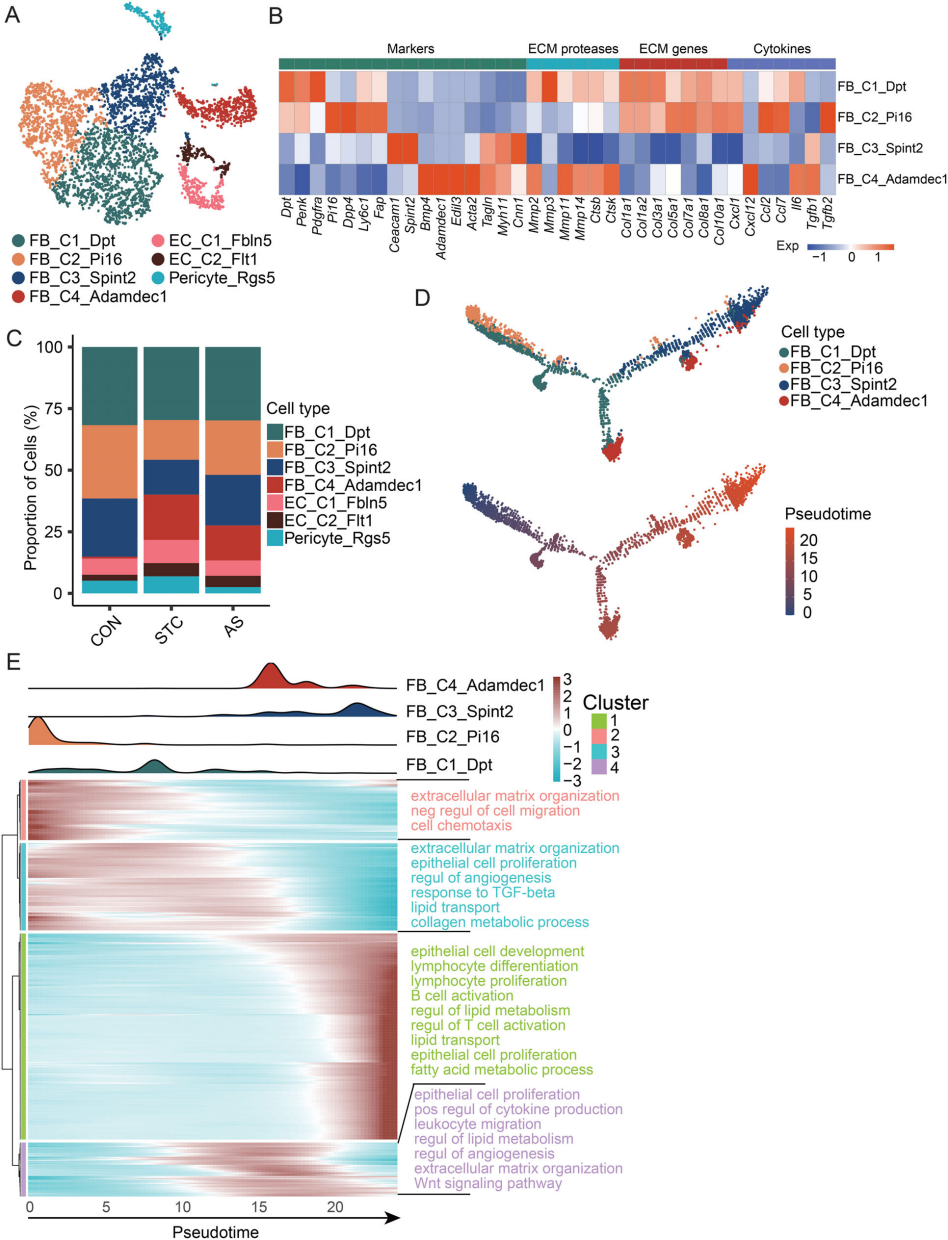
**Dynamic variation in fibroblast subtypes in STC and AS-IV treatment.** (A) The t-SNE plot of stromal cell subtypes; (B) Heatmap displays the expression of selected gene sets in fibroblast subtypes, including marker genes, ECM proteases, ECM genes, and cytokines; (C) The stacked bar chart illustrates the dynamic changes of stromal cell subtypes between the CON, STC, and AS groups; (D) Pseudotime trajectory analysis of fibroblasts. Top: Trajectory is colored by fibroblast subtypes. Bottom: The trajectory is colored by pseudotime; (E) Heatmap showing the dynamic changes in variable gene expression along the pseudotime (bottom). The distribution of fibroblast subtypes during the transition, along with the pseudotime (top). Subtypes are labeled by colors. GO enrichment was performed by the variable genes. ECM: extracellular matrix; CON: Control group; STC: Group of slow transit constipation model; AS: Group of treatment with AS-IV; AS-IV: Astragaloside IV; GO: Gene ontology; Neg: Negative; Regul: Regulation; Pos: Positive.

As the principal mesenchymal cells, fibroblasts were identified as four subtypes (Figure 7A). As reported in the literature [31], FB_C2_Pi16 expresses *Pi16* and other genes, whereas FB_C1_Dpt expresses *Dpt* and other genes. FB_C3_Spint2 expresses *Ceacam1* and *Spint2*, and FB_C4_Adamdec1 expresses *Acta2*, *Adamdec1*, and other genes (Figure 7B). In comparison to the CON group, the proportion of FB_C3_Spint2 decreased in the STC group, while the proportion of FB_C3_Spint2 increased in the AS group, indicating that STC and AS-IV had an impact on FB_C3_Spint2 (Figure 7C). FB_C4_Adamdec1 was almost absent in the CON group, while it was widely distributed in the STC and AS groups. It expresses *Edil3* (Figure 7B), which is a novel angiogenic factor involved in inflammation and angiogenesis [32, 33]. These findings suggest that the functional status of fibroblasts has been modified by STC disease and AS-IV treatment.

We utilized trajectory analysis to investigate the functional differentiation of fibroblasts (Figure 7D). The distribution of fibroblasts varied across the pseudotime in each subtype, which was divided into four clusters (Figure 7E). FB_C3_Spint2 was mainly located at cluster 1, which involves immune cell regulation, fatty acid metabolism, and lipid metabolism, while FB_C4_Adamdec1 was mainly located at cluster 4, which involves the Wnt pathway and angiogenesis.

## Discussion

STC is a multifactorial gastrointestinal disorder that markedly affects individuals’ health and quality of life. To improve therapeutic outcomes, understanding its pathophysiological mechanisms is crucial. This study utilized scRNA-seq and targeted SCFA quantitation profiling of colonic tissues and fecal samples from STC and AS-IV-treated mice. According to our experience, intestinal transit tests can serve as effective indicators for establishing a STC mouse model. We observed significant alterations in the functional repertoire and cell–cell interactions of colonic epithelial cells and tissue microenvironmental cells, along with changes in gut microbiota and metabolites.

The proper functioning of the intestines is closely linked to the maintenance of the intestinal epithelial barrier. This barrier can become compromised in chronic constipation [34]. AS-IV has been shown to improve the dysfunction of the intestinal barrier in different types of diseases [35, 36]. Therefore, we postulated that the therapeutic effect of AS-IV may be partially attributed to its ability to improve the integrity of the intestinal epithelial barrier. Accordingly, we focused on colonic epithelial cells. Based on network pharmacology-based prediction and analysis, we postulated that *Pparγ* serves as the target gene of AS-IV in the treatment of constipation and plays a core role in the PPI network. The literature has reported that *Pparγ* functions as a regulator of lipid and glucose metabolism and can modulate inflammatory processes by interacting directly with NF-κB [22, 37]. Our findings revealed differential expression of *Pparγ* in colonic epithelial cells between groups. Additionally, we examined the fatty acid and energy metabolism of colonic epithelial cells and verified the existence of metabolic abnormalities in the colonic epithelial cells of the STC group, as well as the correlation between metabolic pathways and *Pparγ* expression. Dysregulation of energy metabolism can impair epithelial cell function. According to our study, the functional changes of epithelial cells may have been influenced by changes in butyric acid and intestinal microbiota. Firstly, one of the main energy sources for intestinal epithelial cells is butyric acid [18]. Therefore, a decline in butyric acid levels in the STC group may lead to insufficient energy supply to the colonic epithelial cells. Secondly, butyric acid is a metabolic product of intestinal microbiota [38]. We observed a decrease in lactobacilli in the STC group mice, which is consistent with the decrease in butyric acid.

Moreover, the colonic epithelium has both absorptive and secretory functions that significantly influence defecation, which may be related to aquaporins [20]. Studies have suggested that patients with constipation exhibit intestinal water transport dysfunction [39]. In our study, we also observed changes in water transport and mucus secretion pathways. Aquaporin 3 and aquaporin 8 exhibit high expression in the colon of patients with STC [40]. Consequently, in our study, increased water transport pathways may indicate increased aquaporin expression. Excessive absorption of water in the intestinal cavity will result in the desiccation of intestinal contents, leading to increased excretory difficulty. To summarize, our findings suggest that epithelial cells in STC experience energy metabolism and water metabolism disorders, resulting in intestinal barrier dysfunction that can be ameliorated by AS-IV.

Previous studies have demonstrated the close association between inflammation and gastrointestinal motility disorders. Our results are in agreement with this notion, indicating a notable involvement of inflammation in STC. Nevertheless, there is limited research on the intestinal mucosal immune system in chronic constipation. A study revealed elevated counts of CD3+, CD4+, CD8+, and CD25+ T cells, along with increased intestinal permeability, in patients with functional constipation, implying that the intestinal immune system is abnormal in patients with chronic constipation [17]. In our study, we employed scRNA-seq to investigate the immune cell populations in STC and the effects of AS-IV treatment, with a particular focus on the status of T cells and B cells. Our findings indicate a significant increase in the proportion of Treg cells in STC. Moreover, the trajectory and cell–cell interaction analysis identified the Adgre-Cd55 ligand-receptor interaction as a potential driver of the differentiation of naïve T cells into Treg cells, with the involvement of B cells in this process. AS-IV was found to suppress this differentiation tendency. Previous studies have reported the immunomodulatory effects of AS-IV in various gastrointestinal diseases. For instance, AS-IV has been shown to exert protective effects on Ara-C-induced intestinal mucositis by inhibiting polarization to M1 macrophages through the AKT signaling pathway [41]. Additionally, AS-IV effectively prevented and alleviated ulcerative colitis by modulating Th17/Treg cell homeostasis and anti-oxidative stress [42]. These findings suggest that AS-IV may also exhibit immunomodulatory effects in the treatment of STC.

We not only included epithelial and immune cells but also focused on fibroblasts, which are a critical stromal cell type. Fibroblasts and myofibroblasts in colon tissue play key roles in promoting epithelial growth and repair, regulating immune response, modulating inflammation, promoting fibrosis, and contributing to tumorigenesis and cancer progression. In colitis and inflammatory bowel disease, impaired function of fibroblast subsets leads to compromised epithelial proliferation and maturation, resulting in disease exacerbation [43, 44]. Our findings revealed that the functional status of pre-existing fibroblasts was also altered in STC. Trajectory analysis provided a more comprehensive illustration of the changes in fibroblast function under the influence of the disease.

Our study offers a comprehensive mapping of dynamic changes occurring in various cell types through the application of scRNA-seq technologies. We observed functional alterations in epithelial cells and various cell types within the tissue microenvironment in STC, which were reshaped by the treatment of AS-IV. This provides a new perspective for enhancing our comprehension and management of STC.

## Conclusion

In conclusion, this study significantly contributes to the understanding of scRNA-seq applications in STC. Our research highlights the impact of STC on epithelial cells and the broader tissue microenvironment, revealing how these changes may influence the pathological processes of the condition. Furthermore, we demonstrate the therapeutic efficacy of AS-IV in modulating these cellular abnormalities.

## Supplemental data

## Data Availability

Datasets are available on request: The raw data supporting the conclusions of this article will be made available by the authors, without undue reservation.
